# Insights into the Interactions of Microalgae and Combined Macrolide Antibiotics: Removal Efficiency, Physiological–Biochemical Responses and Transcriptomic Analysis

**DOI:** 10.3390/plants15071128

**Published:** 2026-04-07

**Authors:** Ting Guan, Junzhuang Wu, Guoxin Tang, Feifan Wu, Wei Gao, Shuhan Ren, Wei Li

**Affiliations:** 1 Co-Innovation Center for Sustainable Forestry in Southern China, College of Ecology and Environment, Nanjing Forestry University, Longpan Road 159, Nanjing 210037, China; guan09432023@163.com (T.G.); wjunzhuang@163.com (J.W.); tanggx10@163.com (G.T.); feifanwu@njfu.edu.cn (F.W.); gw2024@njfu.edu.cn (W.G.); renshuhan@njfu.edu.cn (S.R.); 2 National Positioning Observation Station of Hung-tse Lake Wetland Ecosystem in Jiangsu Province, Hongze, Huai’an 223100, China

**Keywords:** microalgae, macrolide antibiotics, combined ecotoxicity, degradation, molecular mechanism

## Abstract

The widespread occurrence of macrolide antibiotics (MLs) in aquatic environments poses potential ecological risks; however, the interactive effects of MLs, especially combined MLs on microalgae and their removal mechanisms, remain poorly understood. This study investigated the removal efficiency, physiological–biochemical responses, and molecular mechanisms of *Chlorella pyrenoidosa* under single and combined exposure to erythromycin (ERY) and roxithromycin (ROX) over 14 days. The results demonstrated that antibiotic removal efficiency was concentration-dependent and higher in low-concentration treatment. The removal rates of ERY (0.15 mg/L) and ROX (0.02 mg/L) reached 100% and 66.86%, respectively. Notably, in the combined low-concentration group, the presence of ROX promoted the degradation of ERY, with the removal being 11.06–14.77% higher than in single treatment. Conversely, in high-concentration combined treatments (1.63 mg/L ERY + 0.5 mg/L ROX), the removal of ERY was inhibited and the removal of ROX was comparable with the corresponding single treatment. High-concentration treatment groups and combined-treatment groups significantly inhibited microalgae growth and total chlorophyll content, modified the chlorophyll composition, and induced severe oxidative stress. Correlation analysis revealed that antibiotic removal was positively correlated with cell density, chlorophyll content, CAT, CYP450, and GST activities while negatively correlated with SOD, ROS, and MDA. Transcriptomic analysis revealed significant disruption of xenobiotic metabolism pathways, photosynthesis-related processes, and DNA replication/mismatch repair pathways. Key genes involved in stress signaling (e.g., *MKK3*, *MPK3*), detoxification (e.g., *CYP97*, *GSTP*), and photosynthesis (e.g., *HemL*) were differentially regulated, providing molecular evidence for the observed physiological responses and removal behaviors. These findings provide valuable insights for the ecological risk assessment of antibiotic mixtures and the development of microalgae-based wastewater treatment technologies.

## 1. Introduction

Antibiotics have played a significant role in human medicine, livestock husbandry, and aquaculture, with their consumption continuously increasing [1]. After administration, the incomplete metabolism of antibiotics in humans and animals leads to their excretion through urine and feces, ultimately contaminating aquatic and terrestrial environments [2,3]. Conventional wastewater treatment technologies, such as the activated sludge process, exhibit limited efficiency in removing antibiotics [4,5]; consequently, antibiotics with varying concentrations are frequently detected in effluents from wastewater treatment plants, as well as in surface water and groundwater worldwide [4,6]. Macrolide antibiotics (MLs) are a key class of antimicrobial agents that play a crucial role in clinical therapy and agricultural farming due to their unique chemical structures and broad-spectrum antibacterial activity. It has been reported that MLs concentrations in WWTP effluents generally range from ng/L to μg/L, occasionally reaching mg/L levels [7]. For instance, the highest reported concentrations of erythromycin (ERY) and roxithromycin (ROX) globally are 3847 ng/L in Spain and 1070 ng/L in China, respectively [8,9]. The presence of residual macrolide antibiotics in the environment poses potential ecotoxicological effects and may lead to the dissemination of antibiotic resistance genes. More concerningly, the co-existence of various antibiotics may further exacerbate these ecological risks.

Microalgae, as primary producers ubiquitously present in aquatic environments, are of critical significance for maintaining the balance of aquatic ecosystems. Under long-term exposure to antibiotics, microalgae may develop the capacity to remove antibiotics by modulating their metabolic activities [10]. Previous studies have confirmed that microalgae can effectively eliminate a variety of antibiotics through biosorption, bioaccumulation, and biodegradation [11,12,13]. For example, *Chlorella sorokiniana* can remove 80% of sulfamethoxazole (SMX), primarily through biodegradation [14]. When microalgae were exposed to ERY at concentrations of 0.1 mg/L, 1.0 mg/L, and 10 mg/L for 21 days, the residual rates of ERY were 4.04%, 6.28%, and 23.53%, respectively [15]. The maximum removal efficiencies of ciprofloxacin by *Chlorella sorokiniana* and *Scenedesmus dimorphus* were 89.43% and 91.73%, respectively [16]. Based on these findings, current research is increasingly focused on developing microalgae-based wastewater treatment technologies [17,18,19]. However, previous studies have predominantly concentrated on the removal of single antibiotics by microalgae, while investigations into the removal efficacy and underlying mechanisms for mixtures of antibiotics remain insufficient.

The unicellular structure of microalgae determines their sensitivity to pollutants. Long-term exposure to antibiotics can affect microalgal growth, photosynthesis, antioxidant systems, and gene expression levels [20]. Mao et al. [21] found that azithromycin at concentrations of 5–100 μg/L inhibited the growth of *Chlorella pyrenoidosa*, impaired algal photosynthesis, induced oxidative damage, reduced cellular energy reserves, and disrupted cell structure. Erythromycin was observed to cause an increase in autofluorescence, a reduction in chlorophyll a content, and hyperpolarization of the mitochondrial membrane potential in *Pseudokirchneriella subcapitata* [22]. Our previous research also revealed that exposure to ERY and ROX upregulated the activities of antioxidant enzymes superoxide dismutase (SOD) and catalase (CAT) and induced malondialdehyde (MDA) accumulation, suggesting the occurrence of oxidative stress in microalgae [23]. It should be emphasized that the physiological, biochemical, and transcriptional changes induced by antibiotic exposure may enhance microalgal resistance to antibiotics, thereby influencing the removal efficiency of antibiotics by microalgae [24]. Given that coexisting antibiotics may exert synergistic or antagonistic effects on microalgae, there are significant differences in the physiological responses of microalgae when exposed to single versus multiple antibiotics [25,26]. Therefore, we hypothesize that the combined action of multiple antibiotics may trigger unique physiological responses in microalgae, which, in turn, may affect the antibiotic removal ability of microalgae.

To test the hypothesis, this study employed two typical MLs, ERY and ROX, as target pollutants to investigate the removal performance and underlying mechanism of *Chlorella pyrenoidosa* under combined antibiotic stress. The removal behavior of single and combined ERY and ROX at varying concentrations by *Chlorella pyrenoidosa* was examined. The physiological and biochemical responses, including growth inhibition, oxidative stress, and metabolic enzyme variations, as well as the relationships between these responses and the removal of antibiotics, were explored to reveal the adaptation of microalgae to combined antibiotic stress. Furthermore, transcriptomic analysis was conducted to decipher the molecular mechanisms underlying the adaptive strategies of microalgae under combined antibiotic stress. This study is expected to provide a scientific basis for the assessment of the ecological risks of MLs and for the development of microalgae-based wastewater treatment technologies.

## 2. Materials and Methods

### 2.1. Chemicals

ERY (CAS 114-07-8) and ROX (CAS 80214-83-1), both with a purity of >98%, were obtained from J&K Chemical Ltd. (Shanghai, China). The reagents used for Blue Green Medium (BG11) were purchased from Sinopharm Chemical Reagent Co. Ltd. (Beijing, China). The components of the BG11 medium are provided in Tables S1 and S2. The fluorescent probe 2′,7′-dichlorodihydrofluorescein diacetate (DCFH-DA, CAS 4091-99-0, ≥98% purity) for reactive oxygen species (ROS) detection was purchased from Shanghai Aladdin Biochemical Technology Co., Ltd. (Shanghai, China). Ultrapure water (>18.2 MΩ) was obtained from the Milli-Q Advantage A10 system (Darmstadt, Germany).

### 2.2. Algal Cultivation

*Chlorella pyrenoidosa* (FACHB-11) was obtained from the Freshwater Algae Culture Collection at the Institute of Hydrobiology, Chinese Academy of Sciences (Wuhan, China). The strain was inoculated in autoclaved (121 °C, 30 min) BG11 medium and cultured in an illumination incubator (MLR-352 H-PC, Panasonic, Osaka, Japan) under the following conditions: temperature of 25 ± 1 °C, light intensity of 200 μmol/(m^2^·s), and a 12 h light/12 h dark cycle. The culture was used for the subsequent experiments once it reached the exponential growth phase.

### 2.3. Experimental Procedure

The experiments were conducted in 250 mL Erlenmeyer flasks containing BG11 medium. A 10 mL aliquot of *Chlorella pyrenoidosa* culture was transferred into each flask to reach an initial cell density of 1 × 10^6^ cells/mL, followed by the addition of ERY and ROX either individually or in combination. The exposure concentrations were set based on previous 96 h acute toxicity data. In the single-exposure assays, ERY concentrations were set at 0.15 mg/L (E1) and 1.63 mg/L (E2), corresponding to EC_10_ and EC_30_ values, respectively. Similarly, ROX concentrations were set at 0.02 mg/L (R1) and 0.5 mg/L (R2), representing its EC_10_ and EC_30_ levels. For the combined toxicity assessments, two binary mixtures (L1 and L2) were prepared, each containing the EC_10_ and EC_30_ concentrations of both antibiotics. In the control group (CK), the antibiotic solutions were replaced with an equal volume of sterilized ultrapure water. The total volume for each treatment group was set at 100 mL. All experiments were performed in triplicate under strictly sterile conditions. Following preparation, the reaction solutions were incubated in an illumination incubator under the same conditions as described above. Samples were collected at the predetermined time intervals (0, 4, 7, 10, and 14 d) for subsequent analysis. Detailed treatment information is presented in Table S3 of the Supplementary Materials.

### 2.4. Analytical Methods

#### 2.4.1. Antibiotic Degradation Analysis

The concentrations of ERY and ROX were determined using high-performance liquid chromatography coupled with mass spectrometry (HPLC-LTQ-Orbitrap-MS, Thermo Scientific, Waltham, MA, USA). Prior to analysis, 2 mL of algal suspension collected from each treatment was filtered through a 0.22 μm membrane filter. The resultant filtrate was then directly injected for analysis. Chromatographic separation was performed on an Agilent EC-C18 column (100 mm × 2.1 mm, 2.7 μm) with a mobile phase consisting of 0.1% formic acid aqueous solution-acetonitrile (60:40, *v*/*v*) at a flow rate of 0.2 mL/min. The injection volume was 5 μL. Mass spectrometry was performed using a heated electrospray ionization (HESI) source in positive ion mode. The key source parameters were optimized as follows: sheath gas flow rate of 40 arb; auxiliary gas flow rate of 10 arb; ion source temperature of 350 °C; and capillary temperature of 325 °C. Voltage parameters were set to a spray voltage of 3.5 kV, a capillary voltage of 9 V, and a tube lens voltage of 100 V. Data acquisition was carried out using a full scan (*m*/*z* 100–1000, resolution 30,000) triggering data-dependent MS/MS acquisition (resolution 30,000). For collision-induced dissociation (CID), the isolation window was set to 1.0 Da, the normalized collision energy was 35%, and the activation time was 30 ms.

#### 2.4.2. Algal Growth

The biomass of algal cells was quantified by measuring the optical density at 680 nm (OD_680_) using a UV-Vis spectrophotometer (Lambda 365, PerkinElmer, Waltham, MA, USA) and a previous established correlation between OD680 and cell density as Equation (1), in which cell density was calculated based on the microalgal cell number determined using a hemocytometer under a microscope.

(1) $${Cell\;density(10}^{6}cells/mL)=21.867\times {OD}_{680}+0.004(R^{2}=0.9911)$$

The specific growth rate was calculated according to Equation (2):

(2) $$\mu =\frac{lnN_{t_{i}}-lnN_{t_{0}}}{t_{i}-t_{0}}$$

where *N_ti_* and *N_t_*_0_ represent the cell density at time *t_i_* and *t*_0_, respectively.

#### 2.4.3. Photosynthetic Pigment Content

Photosynthetic pigments including chlorophyll a, chlorophyll b, and total chlorophyll were quantified following a modified ethanol extraction protocol [23,24]. Briefly, 10 mL of microalgal culture was centrifuged at 8000 rpm for 10 min, and the pellet was resuspended in 10 mL of 95% ethanol. After incubation at 4 °C in darkness for 24 h, samples were centrifuged again at 8000 rpm for 10 min. The absorbance was measured at 665 nm and 649 nm for chlorophyll a/b and total chlorophyll quantification and analysis. Chlorophyll a/b and the total chlorophyll contents were calculated using Equations (3)–(5).

(3) $$\;chl-a\left(mg/L\right)=13.7A_{665}-5.76A_{649}$$

(4) $$chl-b\;\left(mg/L\right)=25.8A_{649}-7.60A_{665}$$

(5) $$\;\;total\;chlorophyll\;\left(mg/L\right)=6.10A_{665}-20.04A_{649}$$

#### 2.4.4. Oxidative Stress and Metabolic Enzyme Analysis

At preset intervals, the microalgal cells were harvested to determine the ROS levels, antioxidant enzyme activities, MDA content, cytochrome P450 (CYP450) content and glutathione S-transferase (GST) activity based on our previous publications [23,24]. ROS levels were measured using the DCFH-DA fluorescent probe assay [24]. SOD activity, CAT activity and MDA content were measured using the xanthine oxidase method, ammonium molybdate method and thiobarbituric acid (TBA) reaction method, respectively [23]. CYP450 content was determined using an enzyme-linked immunosorbent assay, and GST activity was determined using a colorimetric method [24]. All commercial assay kits for SOD, CAT, MDA, CYP450 and GST analysis were purchased from Nanjing Jiancheng Biotechnology Institute (Nanjing, China).

#### 2.4.5. RNA Extraction and Transcriptomic Analysis

The collected algal cells after 14 d of exposure were further used for ribonucleic acid (RNA) extraction and transcriptomic sequencing by Majorbio Bio-Pharm Technology Co., Ltd. (Shanghai, China). After centrifugation at 8000 rpm/min for 5 min, the pellets were immediately flash-frozen in liquid nitrogen and stored at −80 °C for subsequent transcriptomic analysis. Total RNA was extracted using TRIzol reagent kit (Invitrogen, Carlsbad, CA, USA) according to the manufacturer’s protocol. RNA purification, reverse transcription, library construction, and sequencing were performed according to the manufacturer’s instructions (Illumina, San Diego, CA, USA). Then, RNA quality was determined by 5300 Bioanalyser (Agilent, Santa Clara, CA, USA) and quantified using the NanoDrop-2000 (Thermo Scientific, Waltham, MA, USA). Only a high-quality RNA sample (OD260/280 = 1.8~2.2, OD260/230 ≥ 2.0, RQN ≥ 6.5) was used to construct sequencing library. Differential expression analysis was performed using the DESeq2 or DEGseq. Differential expressed genes (DEGs) with ∣log2FC∣ ≥ 1 and FDR < 0.05 (DESeq2) or FDR < 0.001 (DEGseq) were considered to be significantly different expressed genes. Additionally, functional enrichment analyses, including Gene Ontology (GO) and Kyoto Encyclopedia of Genes and Genomes (KEGG) pathway analyses, were performed using Goatools (https://github.com/tanghaibao/Goatools (accessed on 1 March 2026)) and KOBAS (http://kobas.cbi.pku.edu.cn/home.do (accessed on 1 March 2026)), respectively.

### 2.5. Statistical Analysis

All experiments were performed in triplicate. Data visualization was conducted using Origin 2021. Statistical analyses were performed using one-way analysis of variance (ANOVA) followed by Tukey’s post hoc test (SPSS version 28) to assess differences between the control and treatment groups. A probability level of *p* < 0.05 was considered statistically significant.

## 3. Results and Discussion

### 3.1. Degradation of Single and Combined ERY and ROX

**Figure 1:**
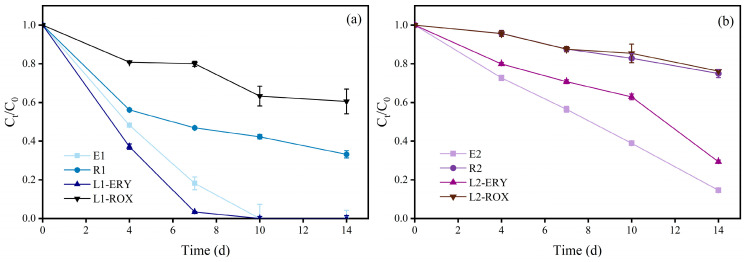
Removal of ERY and ROX in the single and combined treatment system under low (**a**) and high (**b**) concentration treatments. Error bars indicate standard deviation (n = 3).

The removal efficiencies of individual ERY, ROX, and their mixture by microalgae are illustrated in Figure 1. As shown in the figure, the removal rates of both ERY and ROX increased over time and exhibited a clear concentration-dependent pattern, with the removal efficiency in the low-concentration treatment groups being consistently higher than that in the high-concentration groups. Specifically, in the low-concentration treatments, the removal rate of single ERY (0.15 mg/L) increased from 51.80% at 4 d to 100% at 10 d, while that of individual ROX (0.02 mg/L) rose from 43.83% at 4 d to 66.86% at 14 d. Interestingly, in the combined-treatment group, the removal rate of ERY was 11.06~14.77% higher than that in the single ERY treatment during 4~7 d, whereas the removal rate of ROX was lower than that in the single ROX treatment. Theoretically, the additional presence of ROX in the combined treatment enhances the overall toxicity to microalgae, which could lead to a reduction in the removal of both antibiotics. However, the observed increase in ERY removal in the combined treatment suggests that the presence of ROX facilitated the removal of antibiotics by microalgae. This may be attributed to the moderate oxidative stress induced by ROX, which may activate the microalgal defense and degradation mechanisms [27]. Similar results were previously reported by Xiong et al. [26], who reported that the removal rate of sulfamethazine (0.5 mg/L) by *Scenedesmus obliquus* was 15.9% but increased to 51.8% upon the addition of 0.2 mg/L sulfamethoxazole.

In contrast, in the high-concentration treatment groups, the removal rate of ERY in the combined group remained lower than that in the single treatment throughout the 14-day cultivation period. The removal rate of ROX in the combined group was lower than that in the single treatment from 4 d to 10 d but slightly exceeded that of the single treatment at 14 d. The total antibiotic concentration in the combined treatment represents the sum of the concentrations in two individual single treatments, resulting in significantly higher ecotoxicity. Therefore, the lower antibiotic removal observed in the high-concentration combined group is reasonable. The higher removal rate of ROX in the combined-treatment group on day 14 may be attributed to the long-term exposure process. On the one hand, the removal of both antibiotics alleviated their stress on the microalgae; on the other hand, the microalgae gradually developed resistance to the antibiotics during prolonged exposure, thereby exhibiting enhanced removal capabilities.

### 3.2. Responses of Microalgae to Single and Combined ERY and ROX

#### 3.2.1. Microalgal Growth State

**Figure 2:**
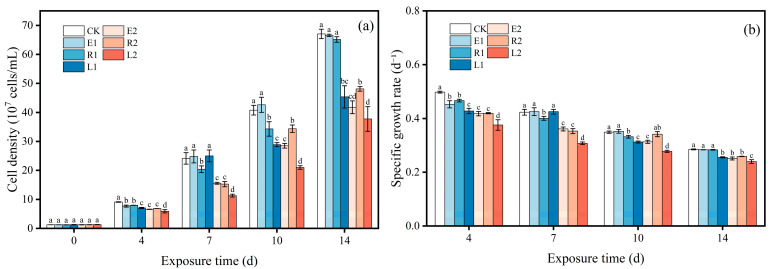
Effect of single and combined ERY and ROX on the cell density (**a**) and specific growth (**b**) of *Chlorella pyrenoidosa*. Error bars indicate standard deviation (n = 3). Different letters above histograms represent significant differences between different treatment groups (*p* < 0.05).

Figure 2 shows the effects of single and combined ERY and ROX treatments on the growth of *Chlorella pyrenoidosa* in terms of cell density and specific growth rate. Overall, in the low-concentration treatment groups, the inhibition rates of cell density by single ERY, ROX, and the combined treatment were −4.56~15.56%, 2.87~15.84%, and −3.69~29.13%, respectively (Figure 2a). The inhibitory effects of antibiotics on the growth of microalgae varied with the cultivation time. ERY, ROX, and their mixture all inhibited the growth of microalgae at 4 d, with the combined treatment exhibiting a higher inhibition rate than the single treatments. However, when the culture time was extended to 7 d, the single ERY and combined treatments promoted microalgal growth, although no significant difference was observed compared to the control group. This may be attributed to the removal of ERY, as the residual concentration in the system had fallen below 20% of the initial concentration; at such reduced levels, ERY may exert a promotional effect on algae [28]. Mao et al. [21] demonstrated that low concentrations (0.5–1 μg/L) of azithromycin induced a typical hormesis effect in microalgae. Similarly, Zhou et al. [29] reported that low concentrations of ofloxacin (OFL, 1~100 μg/L) promoted algal growth by 5.39~9.79%, whereas high concentrations of OFL (1000 μg/L) caused oxidative damage and inhibited microalgal growth. Furthermore, microalgae may assimilate low concentrations of antibiotics as nutrients, thereby facilitating their own growth [30]. Conversely, when the culture time was further increased to 10 and 14 d, the combined treatment exerted a significant inhibition on microalgal growth. This may be due to the toxic effects of long-term antibiotic exposure on the microalgae, or potentially the result of more toxic degradation products generated from ERY and ROX, as well as the complex interactions among these degradation products. Regarding ROX, it significantly inhibited microalgal growth at 4~10 d; however, the inhibitory effect decreased at 14 d, exhibiting no significant difference compared to the control group. This alleviation is attributed to the reduction in ROX concentration due to its removal in the later stages of cultivation.

In the high-concentration treatment groups, the inhibition rates of cell density by single ERY, ROX, and the combined treatment were 27.48~35.49%, 15.66~28.24%, and 35.16~53.15%, respectively. There were no significant differences in the inhibition rates between the single ERY and single ROX treatments at 4~7 d. However, the inhibition rate of the single ROX treatment was lower than that of the single ERY treatment at 10~14 d. This finding is consistent with the increased removal rate of ROX observed in the later stages, where the faster removal rate reduced the ROX concentration in the culture system, thereby alleviating its toxicity to microalgae. Throughout the cultivation period, the inhibition rates of the combined treatment were significantly higher than those of the single ERY and ROX treatments, indicating that the high-concentration antibiotics exhibited greater toxicity. This heightened toxicity explains why the degradation of both antibiotics was inhibited in the combined-treatment group.

#### 3.2.2. Changes in the Chlorophyll Content

**Figure 3:**
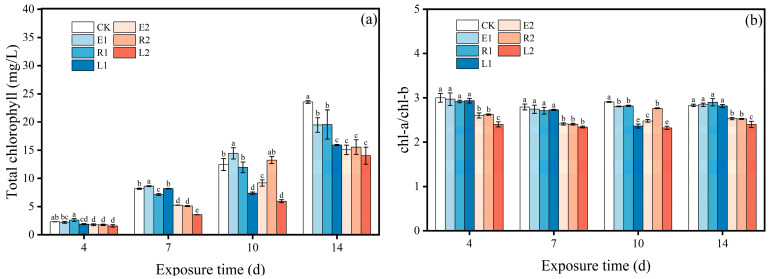
Effect of single and combined ERY and ROX the total chlorophyll (**a**) and Chl-a to Chl-b ratio (**b**) of *Chlorella pyrenoidosa*. Error bars indicate standard deviation (n = 3). Different letters above histograms represent significant differences between different treatment groups (*p* < 0.05).

Chlorophyll, universally present in photosynthetic organisms, serves as a critical pigment for photosynthesis in plants, algae, and certain bacteria. Its unique molecular structure and function play a pivotal role in light energy capture and conversion [31]. Overall, the variation in total chlorophyll content in *Chlorella pyrenoidosa* showed a similar trend with the microalgal growth (Figure 3a). In the low-concentration single ERY treatment groups, the total chlorophyll content at 7 and 10 d was higher than that in the control group, whereas the content at 14 d was lower than that in the control group although no significant difference was observed. Similar results have been reported in previous studies, in which low concentrations of azithromycin led to elevated levels of carotenoids and chlorophyll b in microalgae [21]. The low-concentration combined-treatment group showed no significant difference from the control at 7 d; however, the inhibition rates reached 40.82% and 32.37% at 10 and 14 d, respectively, which were significantly higher than those in the single-treatment groups.

The high-concentration treatment groups significantly inhibited the total chlorophyll content of the microalgae. The total chlorophyll content in the single high-concentration ERY and ROX treatment groups decreased by 36.08% and 33.94% at 14 d, respectively. Moreover, the inhibition in the high-concentration combined-treatment group was significantly higher than that in the single-treatment groups. These results indicate that high concentrations of antibiotics can inhibit chloroplast formation, interfere with protein biosynthesis, and degrade chlorophyll, thereby impairing photosynthetic capacity, cell proliferation, and growth [32]. Notably, the inhibition rate of chlorophyll content in the combined-treatment group peaked at 56.18% at 7 d, decreasing to 52.13% at 10 d and 40.44% at 14 d, respectively. This reflects a certain degree of resistance and adaptive capacity of the microalgae to high-concentration antibiotic stress.

Alterations in the composition and structure of photosynthetic pigments can also reflect damage to the microalgal photosynthetic system under antibiotic exposure [33]. Figure 3b illustrates the variations in the Chl-a/Chl-b ratio across different treatment groups. Throughout the exposure period, the Chl-a/Chl-b ratios in all high-concentration treatment groups were significantly reduced compared to the control group. This suggests that the antibiotics caused an imbalance in the antenna pigment complex ratio or damage to the photosynthetic reaction centers, leading to a decline in photochemical conversion efficiency [34].

#### 3.2.3. Responses of Antioxidative System

**Figure 4:**
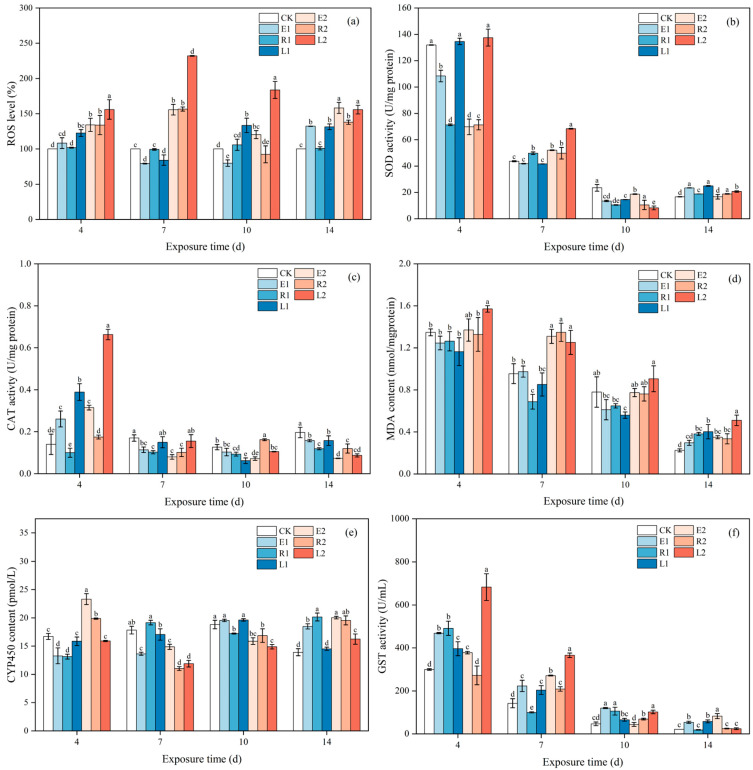
ROS level (**a**), SOD activity (**b**), CAT activity (**c**), MDA content (**d**), CYP450 content (**e**), and GST activity (**f**) of *Chlorella pyrenoidosa* cells under 14 d exposure to single and combined antibiotics. Error bars indicate standard deviation (n = 3). Different letters above histograms represent significant differences between different treatment groups (*p* < 0.05).

When microalgae are subjected to environmental stress, ROS are generated intracellularly. Excessive accumulation of ROS can induce oxidative damage within the microalgae. Upon experiencing certain levels of damage, the algae mobilize their own antioxidant systems to scavenge the produced ROS, thereby maintaining intracellular ROS homeostasis [35]. The production and scavenging of ROS typically exhibit a dynamic equilibrium. Antibiotics can block photosynthetic electron transport in microalgae, forcing electrons to accumulate and react with molecular oxygen, resulting in substantial ROS formation in chloroplasts. This process disrupts the existing intracellular redox balance, triggering oxidative stress in algal cells [31]. In this study, the low concentration single ERY and ROX treatment groups exhibited relatively low ROS levels during 4~10 d, whereas the combined-treatment group generally displayed higher ROS levels (Figure 4a). In contrast, the high-concentration treatment groups displayed elevated ROS levels throughout the entire exposure period, all of which were higher than those in the low-concentration groups, indicating that high-concentration antibiotics induced more severe oxidative stress. Wan et al. [36] reported that cyanobacteria exhibited significantly elevated ROS levels under external stress. Notably, the high-concentration combined-treatment group (L2) displayed the highest ROS levels throughout the exposure period, reaching 155.81~231.99% of the control group, with a peak at 7 d. This trend is consistent with the observed inhibition of chlorophyll content.

Microalgal cells can mitigate oxidative stress by producing antioxidant enzymes such as SOD and CAT to eliminate ROS [37]. SOD serves as the first line of defense, dismutating superoxide anion radicals into H_2_O_2_ and O_2_ [37]. While the enzymatic reaction of SOD can eliminate ROS to a certain extent and alleviate oxidative stress, the H_2_O_2_ produced remains potentially harmful to algal cells. Consequently, the second line of defense, CAT, is activated to further decompose H_2_O_2_ into harmless H_2_O and O_2_ [38], thereby reducing cellular damage. Figure 4b,c illustrate the effects of ERY, ROX, and their mixtures on SOD and CAT activities in microalgae over a 14-day exposure period. Notably, SOD activities in all single-treatment groups (E1, E2, R1, and R2) exhibited significant inhibition, while their CAT activities remained similar to or even higher than those of the control group at 4 d. This phenomenon has been confirmed in previous studies. Li et al. [15] reported that CAT played the primary role in ROS scavenging when microalgae were exposed to ERY, while SOD was suppressed. By 7 d, most treatment groups showed elevated SOD activity (13.93–56.54%). However, by 10 d, all groups except E2 exhibited inhibition of SOD activity (20.46–64.95%). This pattern may reflect an initial upregulation of SOD in response to oxidative stress signals, followed by its consumption during ROS scavenging to mitigate oxidative damage.

In contrast, in the early cultivation stage (4 d), CAT activity in most treatment groups increased relative to the control group. In particular, CAT activities in the combined-treatment groups (L1 and L2) were 277.75% and 473.64% of the control group, respectively, and significantly higher than those in the corresponding single-treatment groups. However, in the later exposure period (7~14 d), CAT activity in all treatment groups began to decline. The decrease in antioxidant enzyme activity under antibiotic stress may be attributed to two primary reasons. On the one hand, the antibiotic stress may be insufficient to induce significant oxidative stress, failing to generate enough ROS to activate antioxidant enzyme activity [39]. On the other hand, excessive antibiotic toxicity may exceed the tolerance threshold of microalgae, leading to disruption of redox homeostasis and consequent functional decline of the antioxidant enzyme system. In this study, given that ROS levels during 10~14 d of cultivation were significantly higher than those in the control group, the reduction in SOD and CAT activities can be attributed to the latter scenario.

MDA is a product of lipid peroxidation and commonly used as a biomarker to assess the level of oxidative damage in microalgal cells [36]. Figure 4d depicts the effects of ERY, ROX, and their mixtures on MDA content in *Chlorella pyrenoidosa* after 14 d of exposure. Notably, from 4 to 10 d, the MDA content in all high-concentration treatment groups was consistently higher than that in their corresponding low-concentration groups. Moreover, the combined-treatment groups exhibited higher MDA levels compared to the single-treatment groups, indicating that high-concentration antibiotics caused more severe oxidative damage. By 14 d, the MDA content in all antibiotic treatment groups was significantly higher than that in the control group. This trend aligns with the concurrently observed high ROS levels and the suppression of SOD and CAT activities, which together suggest an inadequate antioxidant response to counteract the oxidative stress imposed by ERY and ROX, ultimately leading to pronounced oxidative injury. Yang et al. [40] found that MDA content is more sensitive to changes in microalgal biomass, which corroborates the observed growth inhibition in all experimental groups at 14 d.

#### 3.2.4. Vibrations in Metabolic Related Enzymes

In microalgae, CYP450 and GST constitute the core enzymatic systems involved in xenobiotic metabolism, primarily mediating Phase I and Phase II detoxification reactions, respectively [41]. The CYP450 enzyme system belongs to a superfamily of membrane-bound hemoproteins that participate in the oxidation of xenobiotics. During Phase I reactions, CYP450 enhances the water solubility of substrates by catalyzing typical monooxygenation reactions (e.g., hydroxylation and epoxidation), thereby facilitating the degradation of exogenous compounds [42]. In Phase II reactions, GST conjugates glutathione to the products generated in Phase I, promoting their excretion from microalgal cells [43]. Figure 4e,f show the variations in CYP450 content and GST activity in microalgae exposed to ERY, ROX, and their mixtures for 14 days. During the early exposure stage (4 d), the CYP450 content in the low-concentration ERY and ROX treatment groups (E1, R1) decreased by 20.56% and 21.35%, respectively, compared with the control. In contrast, the high-concentration groups (E2, R2) exhibited significant increases of 48.47% and 23.67%. After 14 days of cultivation, CYP450 levels in all single-exposure groups remained significantly higher than those in the control, whereas the combined-treatment groups showed markedly lower CYP450 content compared with the single-treatment groups. Previous studies have indicated that changes in CYP450 activity are closely associated with the removal of sulfamethoxazole in *Chlorella sorokiniana*, suppression of CYP450 reduces the efficiency of sulfamethoxazole elimination [14]. Therefore, the lower CYP450 levels in the combined-treatment groups may partly explain their reduced antibiotic removal rates relative to the single-exposure groups. On the other hand, GST activity across all treatment groups generally displayed an upregulated trend throughout the exposure period, which is conductive to the excretion of antibiotics.

### 3.3. Correlations Analysis Between Microalgal Growth, Physiological–Biochemical Responses, and Antibiotic Degradation Rates

**Figure 5:**
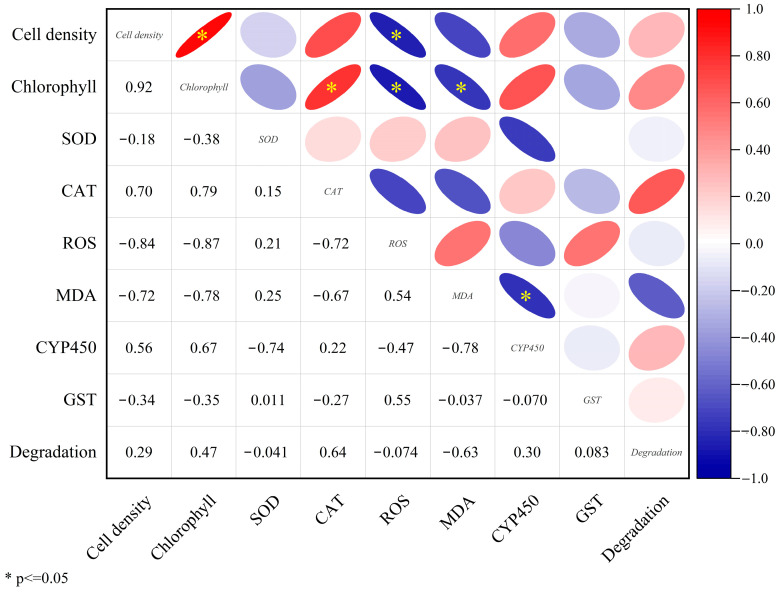
Correlation matrix between cell density, total chlorophyll content, SOD activity, CAT activity, ROS level, MDA content, CYP450 content, GST activity, and antibiotic degradation rate. The color gradient and circle sizes represent the correlation coefficient (r). * represents significant correlation at *p* < 0.05 (bilateral).

To elucidate the interactions between microalgae and antibiotics, a correlation matrix was established to evaluate the relationships among antibiotic effects on microalgal growth and physiological–biochemical parameters (cell density, chlorophyll content, SOD, CAT, ROS, MDA, CYP450, GST) and the removal of antibiotics by microalgae (Figure 5). The antibiotic removal rate showed positive correlations with cell density and chlorophyll content. Previous studies have indicated that an increase in cell density can enhance the removal of macrolides from aqueous environments, primarily through bioadsorption, bioaccumulation, and biodegradation, which are closely associated with microalgal biomass [23]. Meanwhile, lipid peroxidation can inhibit chlorophyll synthesis on thylakoid membranes. It has been reported that photosynthetic pigments such as chlorophyll a, chlorophyll b, and carotenoids can promote antibiotic degradation [44]. Luo et al. [45] also observed that the presence of chlorophyll facilitates the degradation of chlortetracycline and benzopyrene. Antibiotic degradation also showed a significant correlation with CAT activity, which may be attributed to the role of CAT in mitigating oxidative stress and maintaining the physiological–biochemical homeostasis of the microalgae. Antibiotic removal exhibited weak positive correlations with CYP450 content and GST activity, suggesting that besides these two metabolic enzymes, other enzymatic or non-enzymatic mechanisms may contribute to antibiotic metabolism [46]. Conversely, antibiotic removal was negatively correlated with SOD activity, ROS level, and MDA content, especially MDA, which indicates that higher antibiotic toxicity and consequent oxidative damage can impair the ability of microalgae to remove antibiotics.

### 3.4. Transcriptomic Analysis

**Figure 6:**
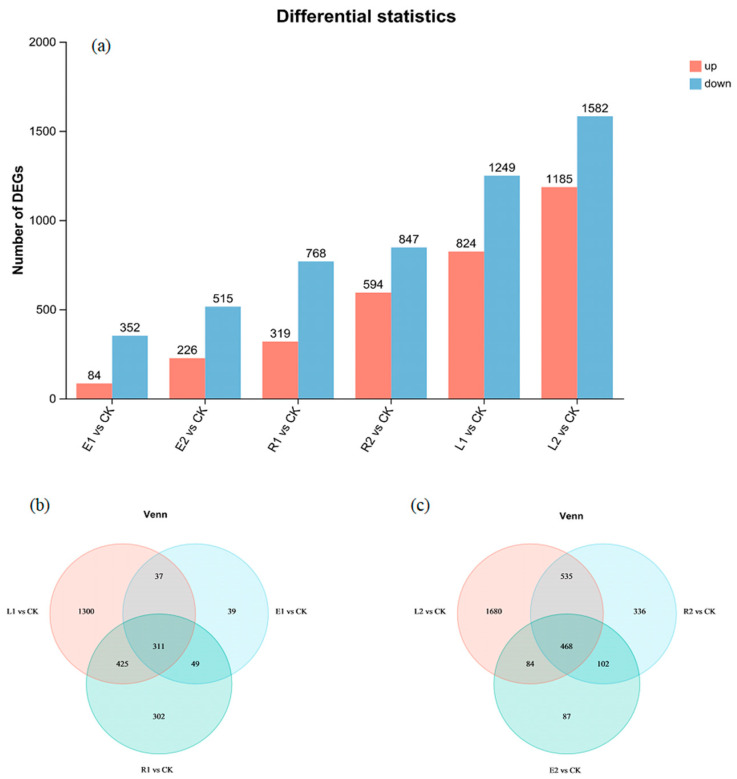
Transcriptomic analysis of the effects of ERY, ROX, and their mixtures on *Chlorella pyrenoidosa*. The number of DEGs in different treatment groups (**a**). Venn diagram of DEGs in the low-concentration and high-concentration treatment groups compared with those in the CK group (**b**,**c**).

To further elucidate the molecular mechanisms underlying the responses of *Chlorella pyrenoidosa* to single and combined ERY and ROX during long-term exposure, RNA sequencing (RNA-seq) was performed on samples from all treatment groups. All samples met the quality control criteria (Table S3) and were suitable for subsequent analysis. Gene expression patterns were highly reproducible within the same treatment but distinct between different treatments (Figure S1). As shown in Figure 6a, exposure to low- and high-concentration ERY and ROX resulted in 436, 741, 1087, and 1441 differentially expressed genes (DEGs), respectively, while their combined treatments induced 2073 and 2767 DEGs. These results indicate that both contaminants significantly affected gene expression in *Chlorella pyrenoidosa*, and the number of DEGs was concentration-dependent. In all groups, the number of downregulated genes exceeded that of up-regulated genes. Venn diagrams revealed 311 common DEGs among the low-concentration groups (E1, R1 and L1), with the L1 group containing the highest number of unique DEGs (1300) (Figure 6b). Among the high-concentration groups (E2, R2, and L2), 468 DEGs were identified, and the L2 group displayed the most unique DEGs (1680) (Figure 6c).

DEGs from each treatment group were subjected to KEGG enrichment analysis (Figure S2) and GO annotation analysis (Figure S3). Based on these analyses, significantly enriched GO categories and KEGG pathways were identified (*p* < 0.05). Among the significantly enriched GO terms (Q-value ≤ 0.05), DEGs from all treatments were prominently associated with categories such as cellular process, metabolic process, binding, catalytic activity, and cellular anatomical entity. These enrichment patterns suggest that antibiotics exert toxicity on *Chlorella pyrenoidosa* by interfering with fundamental cellular processes, metabolic networks, and protein complex functions. This may manifest as damage to organelle structures (e.g., chloroplasts), insufficient energy supply, and the likely activation of detoxification metabolism, including the upregulation of oxidoreductase activity to degrade xenobiotics [47]. Furthermore, KEGG enrichment analysis further identified several pathways commonly and significantly enriched across treatment groups (Q-value ≤ 0.05), including the MAPK signaling pathway, ABC transporters, glutathione metabolism, DNA replication, and fatty acid elongation. This indicates that *Chlorella pyrenoidosa* employs a multi-layered response to external stress, involving MAPK signaling, antioxidant defense, adjustments in energy metabolism, and activation of transport proteins. After entering the cell, contaminants may induce active efflux via ABC transporters to reduce intracellular toxicity. If the contaminant concentration exceeds the efflux capacity of ABC transporters, leading to intracellular accumulation, a burst of reactive oxygen species (ROS) may be triggered. ROS, acting as signaling molecules, can be sensed by the MAPK pathway, thereby activating cellular stress-response mechanisms. Specifically, this involves upregulating genes related to glutathione metabolism to enhance ROS scavenging and maintain cellular homeostasis, while also modulating genes involved in fatty acid elongation and NAD^+^ metabolism to optimize the synthesis and supply of energy substrates, ensuring that energy demands are met under stress conditions [48]. These enriched pathways, together with the physiological and biochemical responses of *Chlorella* to antibiotic exposure discussed earlier, highlight the significant impact of antibiotics on xenobiotic metabolism, photosynthesis, and processes such as DNA replication and mismatch repair in microalgae (Table 1).

#### 3.4.1. Xenobiotic Metabolism-Related Pathways

In eukaryotes, the mitogen-activated protein kinase (MAPK) signaling pathway plays a crucial role in mediating cellular responses to various external stimuli, encompassing both biotic and abiotic stresses. Through this pathway, cells receive diverse signal inputs from the external environment and subsequently regulate their metabolic rates, survival, and proliferation potential, as well as other cellular processes involved in maintaining organismal homeostasis. The MAPK family comprises three main groups: extracellular signal-regulated kinases (ERKs), c-Jun N-terminal kinases (JNKs), and p38 MAPKs. MAPKs are activated by distinct MAP kinases (MAP2Ks), which are, in turn, activated by MAP3Ks [49,50]. In this study, several DEGs enriched in the MAPK signaling pathway were identified, including *MKK3*, *ANP1*, *ERK*, *MPK3*, *MAPK1_2*, and *MAPK1_3*. Specifically, *MKK3* belongs to the MAP2K family and serves as an intermediate kinase connecting upstream MAP3Ks to downstream MAPKs. Upon phosphorylation and activation by upstream MAP3Ks (such as TAK1 and MEKK3), *MKK3* specifically phosphorylates p38 MAPK at Thr180/Tyr182, thereby initiating its activity [51]. In the L1 and L2 treatment groups, *MKK3* expression was upregulated by 0.27-fold and 0.25-fold, respectively, while no significant changes were observed in the single-exposure groups. This suggests that microalgae activate the p38 MAPK pathway to perceive and transmit stress signals under more severe stress conditions, which may partially explain the elevated ROS levels observed in the L1 and L2 groups at day 14. *ANP1*, a plant-specific MAP3K involved in cell division and abiotic stress responses [52], exhibited 0.7-fold downregulation in the L2 group but showed no significant changes in other treatment groups. This indicates that high-concentration combined antibiotic exposure affects cell division, consequently impacting cell proliferation. *MPK3*, belonging to the MAPK family, along with the MAPK family isoforms *MAPK1_2* and *MAPK1_3*, displayed varying degrees of upregulation in the single-exposure groups. *MPK3* serves as a “core executor” in abiotic stress adaptation. Previous studies have demonstrated that *MPK3* and *MPK6* can be activated by elevated ROS levels and function to regulate ROS production and detoxification in response to environmental stress [53].

**Figure 7:**
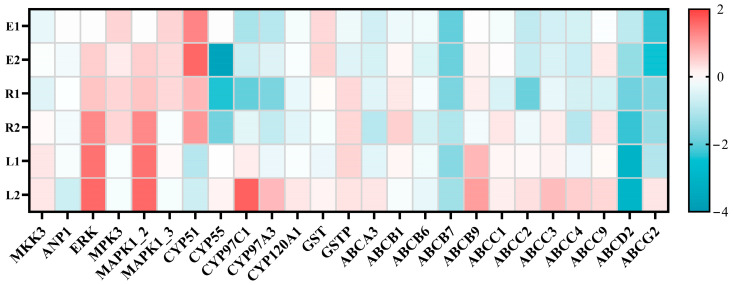
DEGs associated with xenobiotic metabolism.

Upon receiving abiotic stress signals, microalgae induce the activity of intracellular metabolic enzymes. In this study, genes encoding CYP450, including *CYP51*, *CYP55*, *CYP97A3*, *CYP97C1*, and *CYP120A1*, were identified. The CYP450 enzyme family functions as monooxygenases, inserting an oxygen atom into inert hydrophobic molecules to enhance their reactivity and water solubility [24]. *CYP51* constitutes a unique subfamily within the CYP450 superfamily, primarily participating in the sterol biosynthesis pathway. Sterols are essential components of plant cell membranes, critical for maintaining membrane fluidity, stability, and signal transduction. The *CYP51* gene was upregulated in the E1, E2, R1, and R2 treatment groups, with higher upregulation observed at higher concentrations (1.6-fold and 1.08-fold compared to controls). In contrast, the combined-treatment groups (L1 and L2) exhibited downregulation of *CYP51* (0.99-fold and 0.70-fold, respectively) (Figure 7). Suppression of normal *CYP51* gene synthesis disrupts membrane structure and function, ultimately leading to cell death and consequently affecting growth, which accounts for the most pronounced inhibition rates observed in the L1 and L2 groups. Notably, *CYP97* family genes were downregulated in all single-exposure groups but upregulated in the L1 and L2 groups, with the most substantial upregulation observed in the L2 group. Research has suggested that *CYP97* in brown algae may participate in the hydroxylation of β-carotene to zeaxanthin [54], and *CYP97* in *Chlorella pyrenoidosa* may also play a role in antibiotic hydroxylation. Additionally, *CYP120A1*, which is also capable of hydroxylation of pollutants [55], was upregulated in the L2 group. This finding provides a potential mechanistic explanation for the enhanced ROX removal efficiency observed in this combined treatment.

GST constitutes a large enzyme family, with GSTP (typically referring to Phi-class glutathione transferases) representing an important subfamily whose related genes are primarily enriched in the glutathione metabolism pathway. *GST* genes in the E1 and E2 groups exhibited slight upregulation (0.40-fold and 0.14-fold, respectively), and *GSTP* genes in single ROX and combined-treatment groups were also upregulated 0.29~0.44 fold; this is consistent with the GST activity observed at day 14. GST catalyzes the reaction in which glutathione (GSH) combines with electrophilic groups of foreign substances, forming a non-toxic complex [56]. Therefore, the upregulation of *GST* and *GSTP* genes is beneficial for the removal of antibiotics.

ABC transporters function as export or import carriers, being highly involved in transporting nutrients into cells and facilitating the efflux of endogenous toxins and xenobiotic substances [57]. Previous research revealed that ABCC transporters sequester xenobiotic compounds, including glutathione conjugates, chlorophyll degradation products, and heavy metals into isolated vacuoles [58]. Notably, *ABCC* family genes in the L2 group showed marked upregulation, indicating enhanced cellular efflux capacity in the high-concentration combined group, ultimately contributing to increased degradation rates from day 10 to day 14. Notably, *ABCD2* genes were downregulated to varying degrees across all treatment groups, with the most significant downregulation observed in the L1 and L2 groups (3.04-fold and 2.09-fold, respectively). Previous studies have established that fatty acid import into peroxisomes is mediated by *ABCD* family genes [59]. Therefore, the downregulation of *ABCD2* observed in this study may affect fatty acid transport in microalgae, consequently impacting the antioxidant capability and degradation of antibiotics.

#### 3.4.2. Photosynthesis-Related Metabolic Pathways

**Figure 8:**
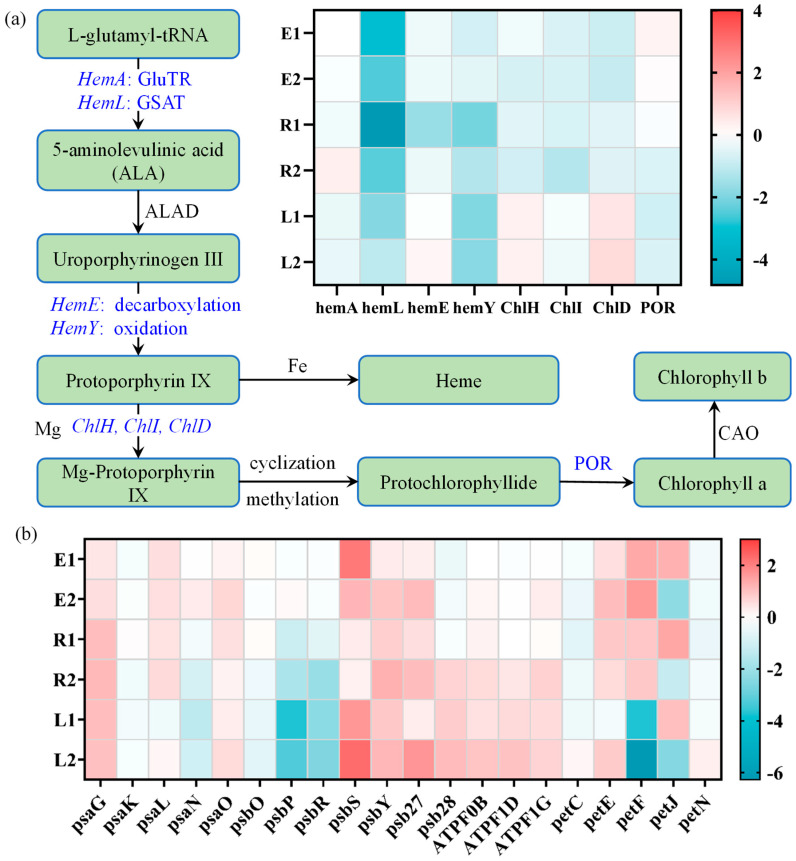
DEGs in the porphyrin and chlorophyll metabolic pathway (**a**) and DEGs in the photosynthetic pathway (**b**).

In photosynthetic eukaryotes such as algae, chlorophyll is essential for light harvesting during photosynthesis and is primarily synthesized through the porphyrin metabolism. Antenna proteins, which include chlorophyll a/b-binding proteins (e.g., LHC I, LHC II), form antenna complexes by binding chlorophyll and carotenoids. These complexes are integral components of photosystem I and II, as well as their associated light-harvesting complexes I and II [60]. The chlorophyll biosynthesis process involves a series of enzymatic reactions that convert porphyrin precursors (e.g., 5-aminolevulinic acid (ALA) and protoporphyrin IX) into chlorophyll a, chlorophyll b, or cytochromes such as those in the cytochrome b6f complex [61]. The gene *hemA* encodes glutamyl-tRNA reductase, which reduces tRNA-bound glutamate to glutamate-1-semialdehyde (GSA), while the protein encoded by *hemL* further catalyzes the conversion of the reduced product GSA to ALA [62]. In most treatment groups, both *hemA* and *hemL* were downregulated (Figure 8a), indicating that the enzymatic reaction converting L-glutamyl-tRNA to ALA was likely inhibited, thereby suppressing the synthesis of all subsequent porphyrin precursors. *HemE* and *hemY* correspond to the decarboxylation and oxidation steps of the porphyrin ring, ultimately affecting the synthesis of protoporphyrin IX. Furthermore, during the formation of protochlorophyllide and chlorophyll a from protoporphyrin IX, genes of the *Chl* family, such as *ChlH* and *ChlD*, were downregulated in the single-treatment groups but upregulated in the combined-treatment groups. In contrast, the activity of protochlorophyllide oxidoreductase (POR), which is responsible for the reduction in protochlorophyllide to chlorophyllide and facilitates chlorophyll a synthesis, was slightly increased in the single-treatment groups but downregulated in both the high-concentration ROX and combined-treatment groups. These changes in gene expression indicate that antibiotics disrupt the chlorophyll biosynthesis pathway, leading to a reduction in chlorophyll content in the treatment groups after 14 days of exposure.

The four multi-subunit membrane protein complexes on the thylakoid membrane, namely, photosystem I (PSI), photosystem II (PSII), the cytochrome b6f complex, and ATPase, constitute the fundamental functional units of the light reactions [63]. In this study, a total of 20 differentially expressed genes (DEGs) related to these complexes were identified (Figure 8b). The *psa*, *psb*, and *pet* gene families encode the protein subunits of PSI, PSII, and the cytochrome b6/f complex, respectively. Studies have shown that the three extrinsic proteins encoded by *psbO*, *psbP*, and *psbQ* form a complex in higher plants, which is crucial for maintaining the structural integrity and oxygen-evolving activity of the PSII oxygen-evolving center (OEC), specifically the Mn_4_CaO_5_ cluster [64]. In the present study, *psbO* and *psbP* were significantly downregulated (0.65–3.71-fold) in the combined-treatment groups, suggesting a reduced oxygen-evolving activity under more severe stress. Additionally, in the combined-treatment groups, certain PSI genes (*psaL*, *psaG*, *psaO*), PSII genes (*psbS*, *psbY*, *psb27*, *psb28*), and the F-type ATPase genes (*ATPF0B*, *ATPF1D*, *ATPF1G*) were upregulated, whereas *petF* in the cytochrome b6/f complex was downregulated. The upregulation of these genes under stress may serve as negative feedback in response to photosynthesis inhibition, a phenomenon consistent with the decrease in the maximum quantum yield of PSII at the physiological level observed in *Raphidocelis subcapitata* [65]. In photosynthetic organisms such as algae, cyanobacteria, and higher plants, the expression regulation of the *petF* gene is closely associated with photosynthetic efficiency. Its aberrant expression may impair the integrity of the electron transport chain, thereby disrupting NADPH generation and photosynthetic rate [47]. Concurrently, the inhibition of photosynthesis induced by combined antibiotics may lead to the excessive generation of ROS, which subsequently triggers oxidative stress (increased levels of SOD, CAT, and MDA) and growth inhibition.

#### 3.4.3. DNA Replication and Mismatch Repair Pathways

**Figure 9:**
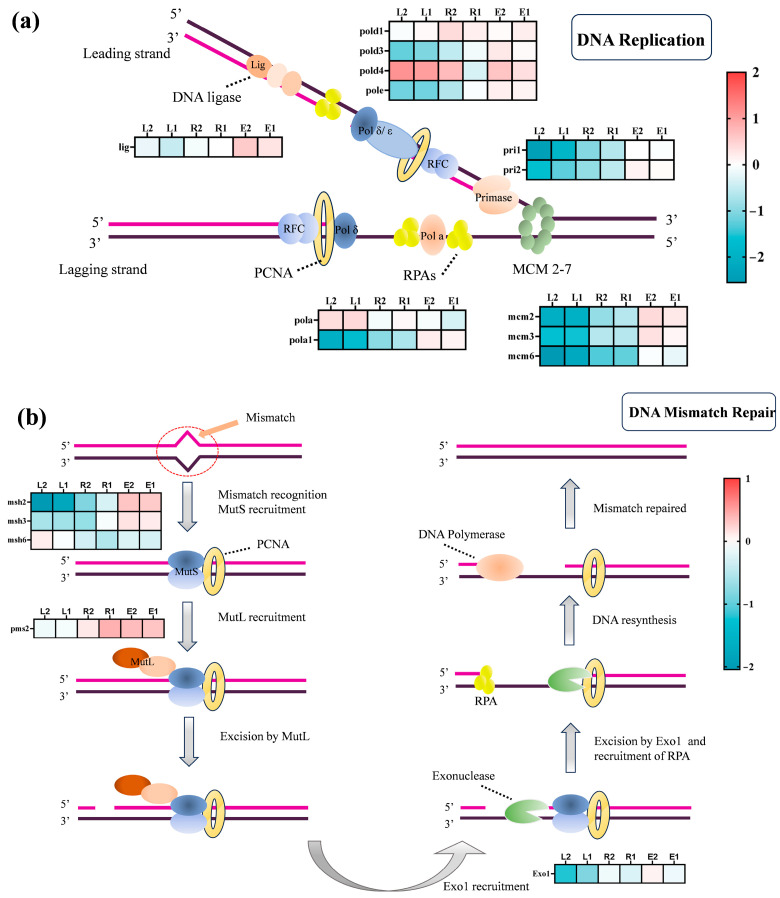
Diagram of DNA replication (**a**) and diagram of DNA mismatch repair (**b**).

DNA replication is a fundamental process for cell proliferation, relying on the coordinated action of multiple proteins, including helicases, single-strand binding proteins, primases, and DNA polymerases [66]. Concurrently, the mismatch repair (MMR) system is responsible for correcting base mismatches generated during replication, thereby maintaining genomic stability and enhancing replication fidelity. In this study, a total of 17 DEGs involved in the DNA replication and MMR pathways were identified (Figure 9). Notably, 13 of these genes exhibited greater downregulation in the single ROX treatment groups (R1, R2) and the combined-treatment groups (L1, L2) compared to the single ERY treatment groups (E1, E2). This indicates that ROX and the combined treatments exert a stronger inhibitory effect on the cell proliferation of *Chlorella pyrenoidosa*. This effect may indirectly lead to a reduced capacity for antibiotic degradation by impairing cell growth.

The RNA primase-encoding genes *pri1* and *pri2*, which are critical components of DNA replication initiation, were significantly downregulated in the ROX and combined-treatment groups. This suggests a substantial impairment of the DNA replication initiation process. Furthermore, the *mcm2-7* helicase complex, responsible for the replication fork formation and DNA unwinding, exhibited more pronounced transcriptional suppression in the combined-treatment groups (downregulated by 1.55–2.21-fold in L1 and 1.61–2.55-fold in L2), indicating a more severe disruption of the replication initiation signals under combined exposure. Studies have shown that the suppression of *mcm2-7* expression can induce DNA damage and genomic instability, thereby disrupting the replication process and ultimately affecting microalgal cell proliferation [28]. On the other hand, proliferating cell nuclear antigen (PCNA), a critical sliding clamp protein in DNA replication and repair, provides processivity for DNA polymerases [65]. The downregulation of *Polα*, *Polδ*, and *Polε*, which serve as the core polymerases for replication initiation, lagging strand synthesis, and leading strand elongation, respectively, collectively reflects the stress state of the DNA replication process. Finally, the downregulation of the DNA ligase (*lig1*) in the ROX and combined-treatment groups further confirms that antibiotic stress inhibits the cell proliferation of *Chlorella pyrenoidosa* by interfering with DNA replication and repair processes.

In the DNA mismatch repair process, the MutS complex scans and recognizes mismatch sites, subsequently recruiting and activating the next core complex MutL to initiate excision and resynthesis steps. In this study, the *msh* genes, key components for mismatch recognition, were suppressed to varying degrees in the ROX and combined-treatment groups. Additionally, the expression of the mismatch repair endonuclease gene *pms2*, a component of the MutL complex, was downregulated in the L1 and L2 treatment groups (0.07-fold in L1; 0.12-fold in L2). The function of the PMS2 protein is to introduce single-strand breaks near the mismatch site, thereby providing an entry point for the exonuclease (*Exo1*) to degrade the mismatched strand [28]. Therefore, combined antibiotics may exert genotoxicity on *Chlorella pyrenoidosa* by disrupting the DNA mismatch repair signaling pathway.

## 4. Conclusions

This study systematically investigated the removal efficiency, physiological–biochemical responses, and molecular mechanisms of *Chlorella pyrenoidosa* under single and combined exposure to ERY and ROX. The results demonstrated that antibiotic removal by microalgae was concentration-dependent, with higher removal efficiencies observed in low-concentration treatments (100% for ERY and 66.86% for ROX). Notably, combined low-concentration exposure enhanced ERY removal by 11.06–14.77% during the early cultivation stage, suggesting that moderate coexisting stress may activate microalgal detoxification mechanisms. However, high-concentration combined exposure significantly inhibited both antibiotic removal and algal growth. Physiological analyses revealed that combined antibiotics induced severe oxidative stress, characterized by elevated ROS levels, MDA accumulation, and disrupted antioxidant enzyme activities. Correlation analysis confirmed that antibiotic removal efficiency was positively correlated with cell density, chlorophyll content, CAT, CYP450, and GST activities, while negatively correlated with SOD activity, ROS, and MDA levels, highlighting the critical role of microalgal physiological status in determining removal capacity. Transcriptomic analysis provided molecular-level insights, revealing that combined exposure induced substantially more differentially expressed genes than single exposures. KEGG enrichment revealed significant disruption of xenobiotic metabolism pathways (MAPK signaling, ABC transporters), photosynthesis-related processes (porphyrin and chlorophyll metabolism, antenna proteins), and DNA replication/mismatch repair pathways. The differential regulation of key detoxification genes (*CYP97*, *GSTP*) and stress signaling genes (*MKK3*, *MPK3*) provided mechanistic explanations for the observed physiological responses and removal behaviors. Overall, this study provides a scientific basis for the ecological risk assessment of antibiotic mixtures in aquatic environments and offers theoretical support for the development of microalgae-based technologies for treating antibiotic-contaminated wastewater.

## Figures and Tables

**Table 1:** A list of key pathways altered by single and combined antibiotic exposure (*p* < 0.05).

Pathway ID	Pathway	Category	Up_Gene	Down_Gene	*p* Value	FDR
**Control vs. E1**						
map04016	MAPK signaling pathway—plant	Signal transduction	—	*YDA*, *copA*, *ctpA*	1.01 × 10^−4^	0.006
map02010	ABC transporters	Membrane transport	*ABCC1*	*ABCB6*, *ABCB7*, *ABCB10*, *ABCG2*	2.58 × 10^−4^	0.008
map00062	Fatty acid elongation	Lipid metabolism	—	*MECR*, *KCS*	6.3 × 10^−4^	0.009
map00480	Glutathione metabolism	Metabolism of other amino acids	—	*GSTP*, *G6PD*, *GST*, *gor*	5.2 × 10^−4^	0.01
map00906	Carotenoid biosynthesis	Metabolism of terpenoids and polyketides	—	*ZDS*, *diox1*, *carT*, *CCD8*	0.001	0.013
map00920	Sulfur metabolism	Energy metabolism	—	*cysE*, *cysJ*, *APR*	0.005	0.043
map00740	Riboflavin metabolism	Metabolism of cofactors and vitamins	—	*ribD*, *ribBA*, *PYRP2*, *ribD*	0.005	0.048
map00760	Nicotinate and nicotinamide metabolism	Metabolism of cofactors and vitamins	—	*nadA*, *nadB*	0.007	0.049
**Control vs. E2**						
map00196	Photosynthesis-antenna proteins	Energy metabolism	*LHCA1*, *LHCA3*, *LHCA4*, *LHCA5*, *LHCB7*	*LHCB1*	7.19 × 10^−5^	0.006
map00480	Glutathione metabolism	Metabolism of other amino acids	*SPE3*	*E1.11.1.11*, *GST*, *GSR*, *GSTP*	2.45 × 10^−4^	0.01
map00906	Carotenoid biosynthesis	Metabolism of terpenoids and polyketides	—	*BCDO2*, *CCD8*, *crtQ*, *diox1*, *crtB*	0.002	0.04
map04016	MAPK signaling pathway—plant	Signal transduction	—	*YDA*, *copA*, *ctpA*	0.002	0.048
**Control vs. R1**						
map00906	Carotenoid biosynthesis	Metabolism of terpenoids and polyketides	—	*LcyB*, *LUT5*, *CCD8*, *BCDO2*, *diox1*, *CYP97C1*	4.88 × 10^−6^	0.0005
map02010	ABC transporters	Membrane transport	*ABCC1*, *ABCD2*	*ABCB6*, *ABCB7*, *ABCB10*, *ABCG2*	0.001	0.0317
map04016	MAPK signaling pathway—plant	Signal transduction	—	*YDA*, *copA*, *ctpA*, *MKK3*	6.6 × 10^−4^	0.0331
map00760	Nicotinate and nicotinamide metabolism	Metabolism of cofactors and vitamins	*E3.1.3.5*, *gabD*	*nadA*, *nadB*	0.001	0.0391
**Control vs. R2**						
map04016	MAPK signaling pathway—plant	Signal transduction	*YDA*	*ERK*, *MAPK1_3*, *copA*, *ctpA*	1.85 × 10^−6^	0.0002
**Control vs. L1**						
map04016	MAPK signaling pathway—plant	Signal transduction	*ERK*	*YDA*, *copA*, *ctpA*, *SNRK2*, *katE*	3.44 × 10^−10^	3.86 × 10^−6^
map03030	DNA replication	Replication and repair	*pold4*, *PCNA*	*pole*, *pola2*, *pold3*, *mcm2*, *mcm3*, *mcm4*, *mcm7*	7.9 × 10^−8^	4.42 × 10^−6^
map00020	Citrate cycle (TCA cycle)	Carbohydrate metabolism	*PDHA*, *PDHB*, *DLD*, *pdhB*, *DLAT*	*SDHB*, *pdhA*, *pdhB*, *por*, *MDH2*, *gltA*	1.18 × 10^−4^	0.0044
map00650	Butanoate metabolism	Carbohydrate metabolism	*gabD*	*adhE*, *bdhA*, *E2.3.1.54*	4.2 × 10^−4^	0.0094
map00010	Glycolysis/Gluconeogenesis	Carbohydrate metabolism	*pdhA*, *pdhB*, *ALDO*, *E5.1.3.15*, *fbp*, *tpiA*, *gapA*, *pyk*, *glk*	*gapN*, *adhE*, *por*, *ALDH*, *E4.1.1.49*	3.6 × 10^−4^	0.01
map00620	Pyruvate metabolism	Carbohydrate metabolism	*pdhA*, *pdhB*, *pyk*	*aceB*, *adhE*, *por*, *ppdK*, *E2.3.1.54*	0.0012	0.022
map00380	Tryptophan metabolism	Amino acid metabolism	*E3.5.1.4*, *KMO*, *DLD*, *E3.5.1.4*	*ALDH*, *atoB*, *katE*, *DLAT*, *DLST*	0.0016	0.025
**Control vs. L2**						
map03010	Ribosome	Translation	*rpsF*, *rplM*, *rpmC*, *rpmA*, *rplC*, *rpsQ*, *rplK*	*RPL13*, *RPL11*, *rplM*, *RPS19*, *RPLP1*, *RPS8*, *rplS*	1.06 × 10^−31^	1.22 × 10^−29^
map04016	MAPK signaling pathway—plant	Signal transduction	*Ndk*, *MAPK1_3*, *MPK1_2*	*YDA*, *copA*, *ctpA*, *SNRK2*, *katE*	5.88 × 10^−8^	3.38 × 10^−6^
map03030	DNA replication	Replication and repair	*LIG1*, *PCNA*	*pole*, *pold1*, *pola2*, *pola*, *mcm4*, *mcm7*	7.59 × 10^−7^	2.91 × 10^−5^
map00620	Pyruvate metabolism	Carbohydrate metabolism	*pdhA*, *pdhB*, *pdhC*, *accA*, *pyk*	*aceB*, *adhE*, *ALDH*, *pckA*, *atoB*,	4.44 × 10^−6^	1.28 × 10^−4^
map00020	Citrate cycle (TCA cycle)	Carbohydrate metabolism	*pdhA*, *pdhB*, *pdhD*, *pyc*	*MDH2*, *SDH1*, *SDH2*, *pckA*, *LSC1*, *LSC2*, *por*	5.77 × 10^−6^	1.33 × 10^−4^
map00010	Glycolysis/Gluconeogenesis	Carbohydrate metabolism	*pdhA*, *pdhB*, *ALDO*, *pyk*	*gapN*, *adhE*, *por*, *pckA*, *GAPDH*, *ACSS1_2*	2.85 × 10^−5^	5.47 × 10^−4^
map00906	Carotenoid biosynthesis	Metabolism of terpenoids and polyketides	*crtP*, *crtQ*, *crtH*, *LUT1*	*carT*, *CCD8*, *crtB*, *crtZ*	2.7 × 10^−4^	0.0044
map00380	Tryptophan metabolism	Amino acid metabolism	*KMO*, *E3.5.1.4*, *pdhD*	*ALDH*, *atoB*, *katE*, *PAH*, *DLAT*, *DDC*	6.55 × 10^−4^	0.0094
map00650	Butanoate metabolism	Carbohydrate metabolism	*gabD*, *GLYR*	*adhE*, *por*, *bdhA*, *E2.3.1.54*, *paaH*	0.001	0.0136
map00053	Ascorbate and aldarate metabolism	Carbohydrate metabolism	*DHAR*, *adh*, *IMPL2*, *GLDH*	*E1.11.1.11*, *ALDH*, *E1.6.5.4*, *VTC2_5*	0.0015	0.0169
map00970	Aminoacyl-tRNA biosynthesis	Translation	*glnS*, *trpS*, *ileS*, *proS*, *glyQS*, *DARS2*, *metG*, *serS*, *tyrS*	*thrS*	0.0043	0.0445

## Data Availability

The original contributions presented in this study are included in the article/Supplementary Materials. Further inquiries can be directed to the corresponding author.

## Supplementary Materials

The following supporting information can be downloaded at https://www.mdpi.com/article/10.3390/plants15071128/s1, Figure S1: Heatmap of gene clustering between 21 samples; Figure S2: KEGG enrichment analysis treated with ERY and ROX and their mixtures; Figure S3: Analysis of GO annotations under the processing of ERY and ROX and their mixtures; Table S1: Compositions of the BG11 medium; Table S2: Components of A5 trace mental solution; Table S3: Summary of the transcriptome sequencing data.
