# Supplementary material for: Insights into the Interactions of Microalgae and Combined Macrolide Antibiotics: Removal Efficiency, Physiological–Biochemical Responses and Transcriptomic Analysis
Source: Plants (Basel). 2026 Apr 7;15(7):1128. doi: 10.3390/plants15071128 (PMC13074270; doi:10.3390/plants15071128)
Supplement: Supplementary file 1 [file plants-15-01128-s001.zip › plants-4207965-supplementary.pdf]

Supplementary Materials

Insights into the Interactions of Microalgae and Combined Macrolide Antibiotics: Removal Efficiency, Physiological–Biochemical Responses and Transcriptomic Analysis

Ting Guan [1,2], Junzhuang Wu [1,2], Guoxin Tang [1,2], Feifan Wu [1,2], Wei Gao [1,2], Shuhan Ren [1,2] and Wei Li [1,2,*]

[1] Co-Innovation Center for Sustainable Forestry in Southern China, College of Ecology and Environment, Nanjing Forestry University, Longpan Road 159, Nanjing 210037, China; guan09432023@163.com (T.G.); wjunzhuang@163.com (J.W.); tanggx10@163.com (G.T.); feifanwu@njfu.edu.cn (F.W.); gw2024@njfu.edu.cn (W.G.); renshuhan@njfu.edu.cn (S.R.)

[2] National Positioning Observation Station of Hung-tse Lake Wetland Ecosystem in Jiangsu Province, Hongze, Huai'an 223100, China

* Correspondence: liwei@njfu.edu.cn

Table S1. Compositions of the BG11 medium.

Composition	Application quantity	Reserve liquid concentration
$NaNO_3$	10 mL/L	15 g/100 mL
K_2HPO_4	10 mL/L	2 g/500 mL
$MgSO_4{\cdot}7H_2O$	10 mL/L	3.75 g/500 mL
$CaCl_2{\cdot}2H_2O$	10 mL/L	1.8 g/500 mL
$C_6H_8FeNO_7$	10 mL/L	0.3 g/500 mL
$C_6H_8O_7$	10 mL/L	0.3 g/500 mL
$C_{10}H_{14}N_2Na_2O_8$	10 mL/L	0.05 g/500 mL
Na_2CO_3	10 mL/L	1.0 g/500 mL
A5（trace metal solution）*	1 mL/L	——

Table S2. Components of A5 trace mental solution.

Composition	concentration
H_3BO_3	2.86 g/L
$MnCl_2 \cdot 4H_2O$	1.86 g/L
$ZnSO_4 \cdot 7H_2O$	0.22 g/L
$Na_2MoO_4 \cdot 2H_2O$	0.39 g/L
$CuSO_4 \cdot 5H_2O$	0.08 g/L
$Co\ (NO_3)_2 \cdot 6H_2O$	0.05 g/L

Table S3. Summary of the transcriptome sequencing data.

Sample	Clean reads	Clean bases	Error rate (%)	Q20(%)	Q30(%)	GC content (%)
CK1	44885800	6718267218	0.0124	98.49	95.37	61.6
CK2	44219134	6617998544	0.0125	98.4	95.1	62.52
CK3	45307440	6766675676	0.0124	98.47	95.33	62.34
E1_1	44972212	6727677377	0.0125	98.43	95.19	62.2
E1_2	46035916	6883857243	0.0124	98.49	95.36	61.44
E1_3	43307294	6461242458	0.0124	98.47	95.31	61.35
E2_1	40442482	6045785511	0.0125	98.41	95.14	62.24
E2_2	44765490	6695239891	0.0126	98.38	95.04	62.21
E2_3	42430542	6340190486	0.0125	98.44	95.22	61.34
R1_1	44768500	6679691339	0.0125	98.42	95.14	62.18
R1_2	44357860	6647585877	0.0126	98.37	94.99	62.11
R1_3	45748744	6853350387	0.0124	98.47	95.29	61.78
R2_1	43131846	6456092775	0.0125	98.4	95.13	62.14
R2_2	47632266	7127005140	0.0124	98.47	95.34	62
R2_3	45803596	6861528549	0.0124	98.45	95.25	61.51
L1_1	44745336	6700249939	0.0124	98.46	95.28	61.52
L1_2	45563134	6815612834	0.0124	98.45	95.25	61.95
L1_3	49922404	7463678448	0.0124	98.48	95.27	60.9
L2_1	44003232	6577690453	0.0124	98.46	95.29	61.64
L2_2	46438248	6937125560	0.0125	98.45	95.24	61.03
L2_3	45086160	6734867021	0.0125	98.42	95.14	61.44

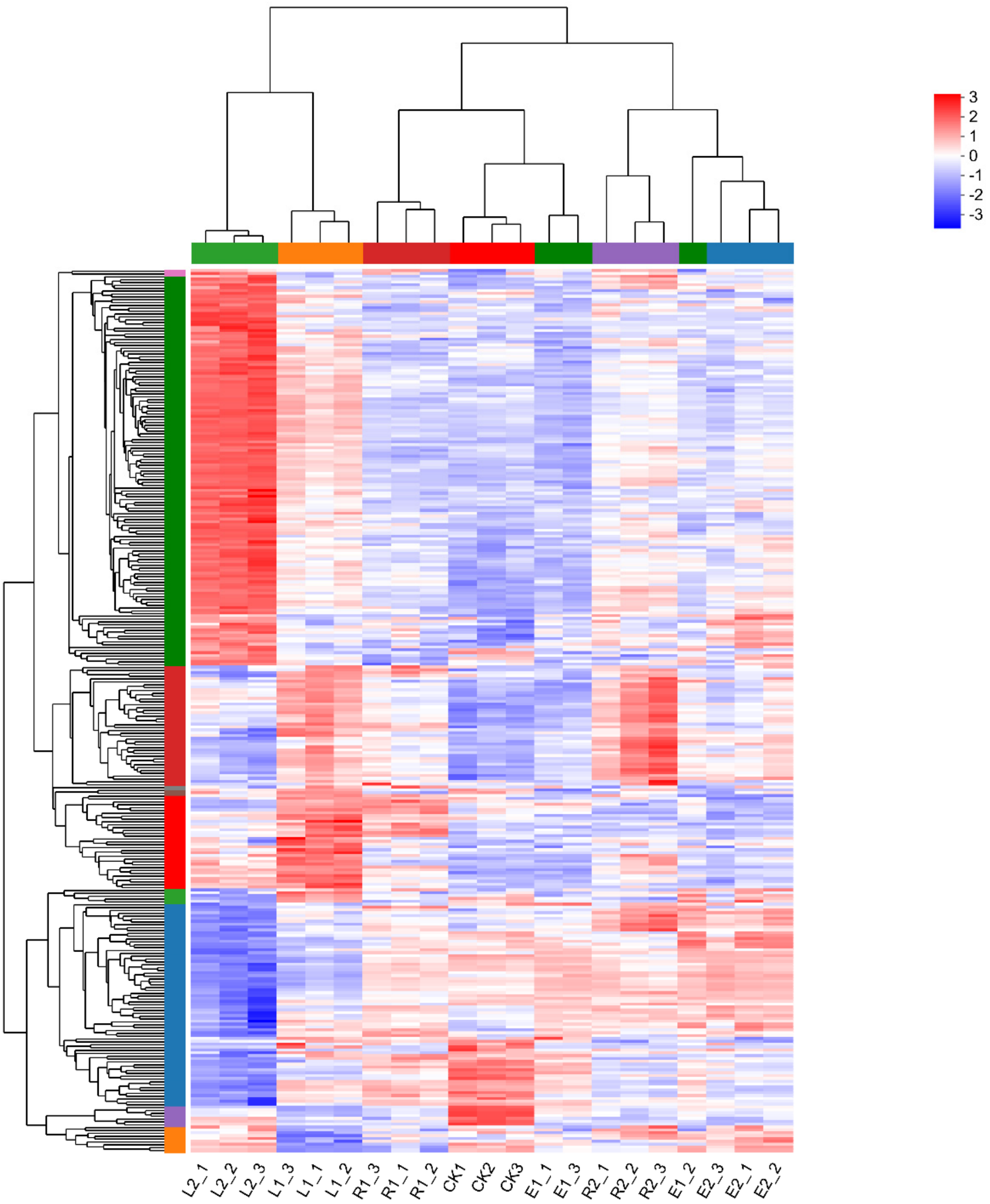

Figure S1. Heatmap of gene clustering between 21 samples.

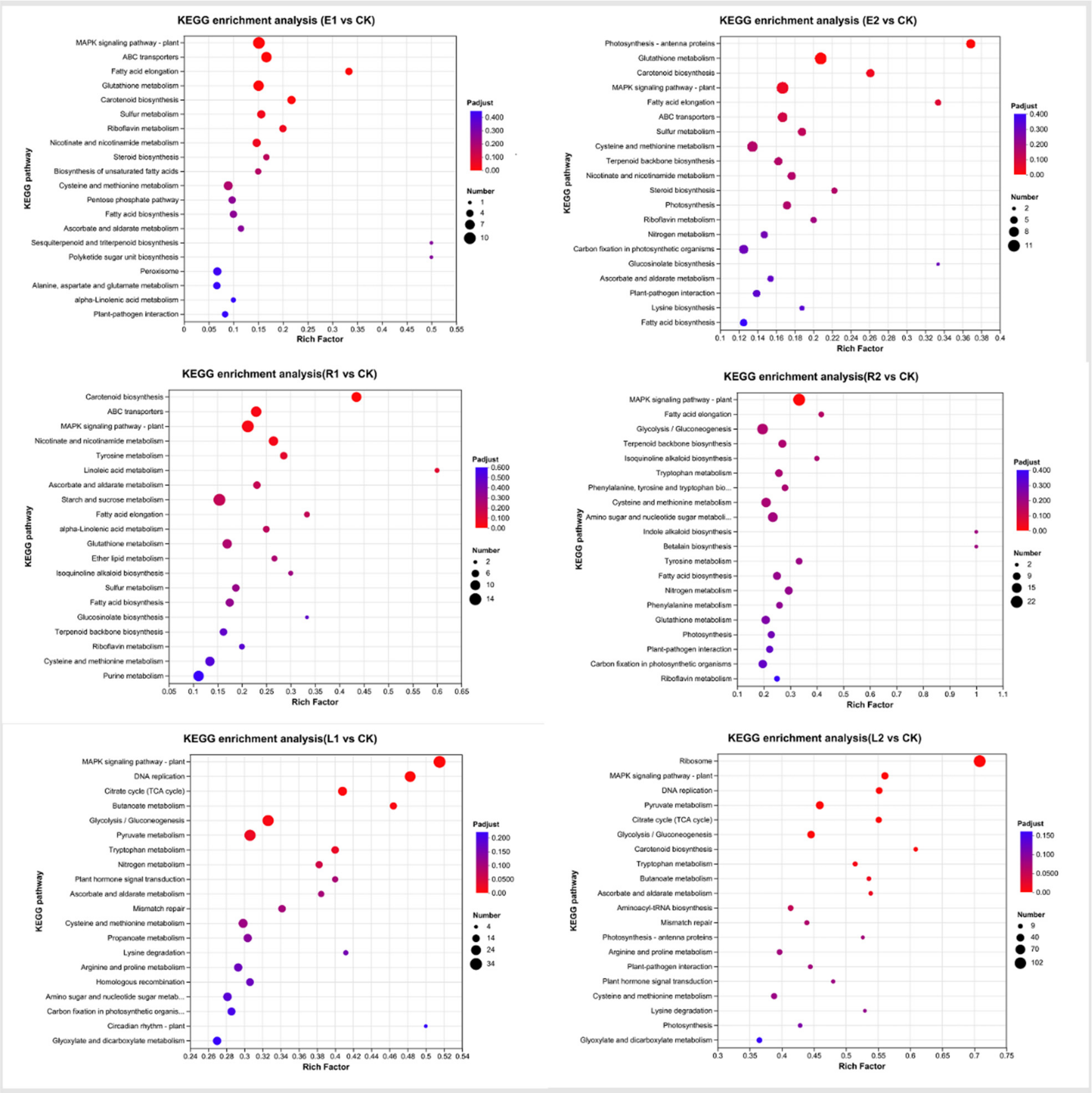

Figure S2. KEGG enrichment analysis treated with ERY and ROX and their mixtures.

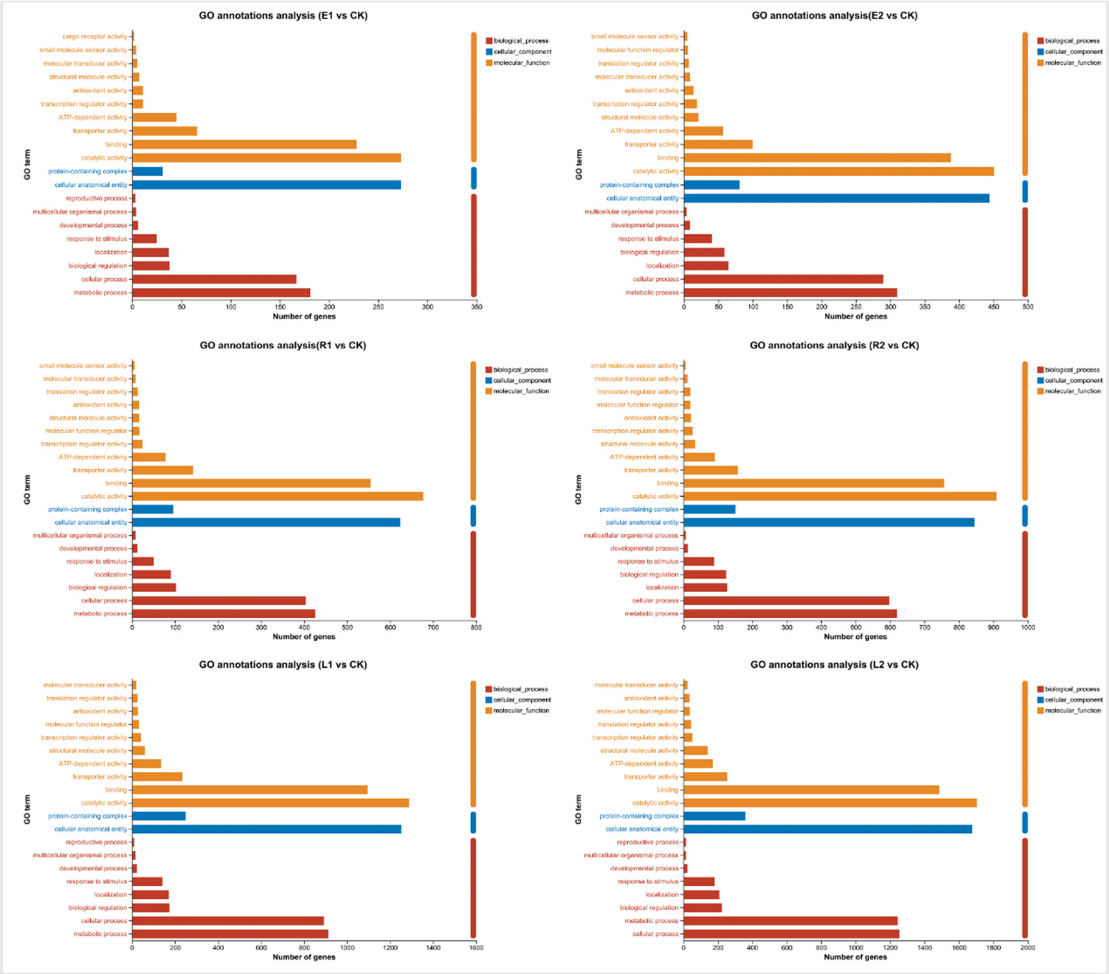

Figure S3. Analysis of GO annotations under the processing of ERY and ROX and their mixtures.
